# Exosome Cell Origin Affects *In Vitro* Markers of Tendon Repair in Ovine Macrophages and Tenocytes

**DOI:** 10.1089/ten.tea.2022.0185

**Published:** 2023-05-10

**Authors:** Devin von Stade, Melinda Meyers, James Johnson, Ted T. Schlegel, Anthony Romeo, Daniel Regan, Kirk McGilvray

**Affiliations:** ^1^ Department of Mechanical Engineering, Orthopaedic Bioengineering Research Laboratory, Colorado State University, Fort Collins, Colorado, USA.; ^2^ Department of Clinical Sciences, Animal Reproduction and Biotechnology Laboratory, Colorado State University, Fort Collins, Colorado, USA.; ^3^ American Orthopedic Partners, Denver, Colorado, USA.; ^4^ Shoulder Elbow Sports Medicine, Chicago, Illinois, USA.; ^5^ Department of Microbiology, Immunology, and Pathology, Flint Animal Cancer Center, Colorado State University, Fort Collins, Colorado, USA.

**Keywords:** tendon, exosome, extracellular vesicle, mesenchymal stromal cell, tenocyte, macrophage

## Abstract

Tendon injuries and disease are resistant to surgical repair; thus, adjunct therapies are widely investigated, especially mesenchymal stromal cells (MSCs) and, more recently, their extracellular vesicles (MSCdEVs), for example, exosomes. Thought to act on resident and infiltrating immune cells, the role of MSCdEVs in paracrine signaling is of great interest. This study investigated how MSCdEVs differ from analogs derived from resident (tenocyte) populations (TdEV). As macrophages play a significant role in tendon maintenance and repair, macrophage signaling was compared by cytokine quantification using a multiplexed immunoassay and tenocyte migration by *in vitro* scratch-wound analysis. TdEV-treated macrophages decreased IL-1 and increased MIP-1 and CXCL8 expression. In addition, macrophage signaling favored collagen synthesis and tenocyte bioactivity, while reducing proangiogenic signaling when TdEVs were used in place of MSCdEVs. These *in vitro* data demonstrate a differential influence of exosomes on macrophage signaling, according to cell source, supporting that local cell-derived exosomes may preferentially drive healing by different means with possible different outcomes compared to MSCdEVs.

**Impact Statement:**

Adipose-derived mesenchymal stromal cell (AdMSC) exosomes (EVs) can improve tendon mechanical resilience, tissue organization, and M2 macrophage phenotype predominance in response to tendon injury. This active area of investigation drives great interest in the function of these exosomes as adjunct therapies for tendon disease, particularly rotator cuff tendinopathy. However, little is known about the effects of EVs as a function of cell source, nor regarding their efficacy in preclinical translational ovine models. Herein we demonstrate a differential effect of exosomes as a function of cell source, tenocyte compared to AdMSCs, on macrophage signaling and tenocyte migration of ovine cells.

## Introduction

Rotator cuff tendinopathy (RCT) is a chronic, debilitating disease resistant to classic interventions, including surgical and nonsurgical treatments/repairs.^1,2^ The underlying pathogenesis is a combination of intrinsic and extrinsic factors, including anatomic predisposition to trauma and poor circulation, combined with cumulative degenerative changes related to age, genetics, and use.^1,3^ RCT manifests as tissue changes, including alterations to tendon fiber arrangement, tendon-bone composition, and changes in tendon microenvironment, cell composition, and cytokine expression.^3,4^ Recent data suggest that cell-to-cell communication during the acute and chronic stages of RCT drives the severity of tendon changes.

In health, tendon fibrocytes (tenocytes) form neatly organized arrays: networks of cell–cell junctions with low levels of connexins allowing for controlled, direct cell-to-cell communication.^5,6^ When cell–cell junctions are lost and/or disrupted in injury, cells that have maintained cell–cell junctions move in cohesive aggregates to fill and “heal” the wound.^7^ While this method of collective cell migration has been studied in greater detail in epithelial cells, cancer, and *in vitro* cultures of mesenchymal stromal cells, less is known about the role of direct, contact-based cell communication, and loss thereof, in the pathogenesis of paucicellular tissue degeneration like RCT.

It is thought that loss of cell-to-cell communication increases the extrinsic influence of paracrine signaling from non-tenocyte-resident and recruited cells of the degenerated tendon microenvironment (e.g., macrophage paracrine signaling), particularly in adult tendons where tenocyte–tenocyte junctions are less frequent.^6,8^ In addition, in the setting of acute injury such as rotator cuff tears, an inevitable sequela of RCT, the sparsely cellular and poorly vascular tendon tissue is further remodeled by a rapid influx of cells due to hemorrhage, immune cell infiltration, and angiogenesis.^9^

Macrophages are recruited early during tendon injury and persist preferentially longer than other recruited immune cells.^10,11^ These cells are crucial to the removal of damaged tissue(s) and cellular debris; however, if macrophage response exceeds this demand, there is increased replacement of the pre-existing tendon by scar tissue.^10,11^ As in other chronic inflammatory conditions such as atherosclerosis, the preferential polarization of these macrophages toward an anti-inflammatory (M2) state over a proinflammatory (M1) phenotype in damaged tendons may drive effective tendon regeneration and healing.^11,12^ The role of paracrine signaling from immune cells, such as macrophages, in the structural function of injured tendons, has driven interest in the complex heterogeneity of cell–cell interactions that may drive the progression of RCT. Extracellular vesicles (EVs) are such means of communication and thus have become of significant research interest as means to control local microenvironments.^13^

EVs have recently emerged as critical regulators of intercellular communication due to their ability to influence target cell function through the transfer of significant payloads of biological cargo, including protein and nucleic acid.^14,15^ Most, if not all, cell types can release EVs, which influence host immune response in a wide range of pathologies including cancer, cardiovascular disease, and viral infections.^14,16^ In several musculoskeletal disorders, adipose-derived mesenchymal stromal cell (AdMSC) EVs are a primary paracrine effector of improvements in tissue healing.^12,15,17–19^ Recently, AdMSC-derived EVs have improved tendon mechanical resilience, tissue organization, and M2 macrophage phenotype predominance in response to tendon injury.^12^ Similarly, in rat models, exosomes from tendon-derived mesenchymal stromal cells have improved tendon healing.^20^

While the underlying mechanism of EV-mediated repair is not fully understood, data suggest both a direct effect on tenocyte bioactivity and an indirect impact through immunomodulatory effects on cytokine expression, particularly through macrophage phenotypic shift (i.e., macrophage “education”).^11,12,21^ For example, in a rat model of Achilles tendon injury, treatment with EV-educated macrophages decreased granulation tissue in injured tendons and improved biomechanics.^11^ As these EVs can have a wide range of effects in different pathologies, the cell source and the microenvironment thereof can influence both the biological cargo of EVs and surface signals that dictate their target cellular tropism.^16^ While AdMSC-derived exosomes (MSCdEV) and tendon-stem cell-derived exosomes have been reported to improve tendon healing in animal models, the underlying mechanisms mediating this healing are not fully understood.^12,20^ Likewise, the potential differences between these mechanisms and that of fully differentiated tenocyte-derived exosomes (TdEV) are not yet known.

As tendon healing is driven by a balance between the clearing of damaged tissue by macrophages and tissue deposition by tenocytes, it is logical that there is a controlled form of communication between resident tenocytes and macrophages. We hypothesize that TdEVs, compared to MSCdEVs, will preferentially drive paracrine, proinflammatory and anti-inflammatory, collagen modulating and angiogenic cytokine expression of macrophages toward a tenogenic phenotype, as well as directly increase tenocyte migration. Thus, TdEVs may preferentially drive tendon healing through their autocrine and paracrine effects.

## Materials and Methods

### Animals

Paired isolations of tenocytes and AdMSCs were harvested (postmortem interval <2 h) from a skeletally mature adult (*n* = 1; 3–4 years old; *Ovis aries*) ewe with no known clinical comorbidity, which was euthanized for an unrelated research study. Peripheral blood-derived monocytes were isolated from blood drawn from ewes (*n* = 3) during routine intake screening with no known comorbidity, collected in ethylenediaminetetraacetic acid coated 10 mL tubes. Collection and use of blood from ewes were approved by the Colorado State University Research Integrity and Compliance Review Office, Institutional Animal Care and Use Committee (IACUC; No. 1503) (Supplementary Fig. S1).

### Isolation and culture of tenocytes and AdMSCs

Subcuticular adipose tissue (2–3 cm^3^) was collected from the shoulder. The infraspinatus tendon was carefully isolated, and a 10 × 5 × 5 mm section of the central tendon body core was aseptically collected. The same procedure was then used to isolate cells from each tissue. These tissue sections were digested in 35 mL of 1:1 Dulbecco's Modified Eagle's Medium with Nutrient Mixture F-12 Ham with 15 mM HEPES and sodium bicarbonate (DMEM:F12, D6421; Sigma-Aldrich) with 200 units penicillin, 0.2mg/mL streptomycin, and 0.5mg/mL amphotericin B (Abx, A5955; Sigma-Aldrich) and 1mg/mL of type 1 collagenase (BP2649; Fisher Bioreagents) for 24 h at 37°C with 5% carbon dioxide under constant agitation as a modification of traditional enzymatic cell isolation.^22–24^

This digesta was then filtered at 100 μm (Cell Strainer, Z742101; Sigma-Aldrich), and the flow through was diluted to 50 mL with Dulbecco's phosphate buffered saline (PBS; D1408; Sigma-Aldrich), centrifuged at 400 *g* for 10 min, and the cell pellet was resuspended in and washed twice in 10 mL of PBS. The resulting cell pellet was resuspended in DMEM:F12 with 10% Fetal Bovine Serum (97068-085, Avantor) and Abx (complete DMEM [CD]) and grown to confluence in a 75 cm^2^ culture flask (156499; Thermo Fisher Scientific) before passage. Cells in passages 2–3 were used for EV purification to limit the phenotypic shift of isolated cells and thus ensure minimal variability in exosome content.^24,25^

Tenocytes were characterized by visual assessment for spindle morphology, and Western blot analysis of 20 μg of cell lysate-derived protein for tenomodulin (TNMD; SAB2108237; Sigma-Aldrich) and Tenascin C (TNC; sc-25328; Santa Cruz Biotechnology). AdMSCs were characterized by trilineage differentiation using Mesenchymal Stem Cell Chondrogenic, Osteogenic, and Adipogenic Differentiation Media (C-28012, C-28013, and C-28016; Millipore Sigma), the results of which were assessed using Alcian blue, Von Kossa, and Oil Red O staining for cartilage, bone, and adipose, respectively.

### EV purification

Both cell lines were amplified in passages 2 and 3 into 225 cm^2^ culture flasks (159934; Thermo Fisher Scientific). While at 80–100% confluence, culture media were aspirated, cells were washed with 5 mL of PBS, and flasks were filled with 15 mL of serum-free DMEM:F12 with Abx. After 24 h, cell-enriched culture media were collected, cell debris was removed by centrifugation at 700 *g* for 10 min, and supernatants were stored at −20°C. Cultures were then “rested” for 24 h in CD. After reaching 100% confluence for at least 24 h, cultures were split and passaged.

Cell-enriched media were pooled within individual cell lines and filter concentrated (Centricon 70; UFC710008; Millipore Sigma). Then, EVs were isolated by size exclusion chromatography (qEV columns; QiZON, qEVoriginal). Fraction 3, the fraction of the highest particle density per EV quantification, was used for all experiments, unless otherwise noted.

### EV quantification

The ZetaView QUATT 4 nanoparticle tracking analysis instrument (NTA) (ZetaVIEW software ver. 8.05.12 SP1; Particle Metrix GmbH) was used to determine the size and concentration of isolated EVs before use. Utilizing the scatter mode of the NTA, the concentration of all EVs was measured. Assessment of the Brownian motion determined individual particle size and concentration. Sample aliquots were diluted in PBS to an average count per frame of 50–500 particles. Based on these measurements, EV concentrations were standardized using PBS as a diluent to 1 × 10^7^ particles/30 μL.

### Western blot

EVs were concentrated from fraction 2 using ultracentrifugation at 110K *g* for 90 min, supernatants were aspirated, and EV pellets were resuspended in radioimmunoprecipitation assay (RIPA) lysis buffer (89901; Thermo Fisher Scientific) with protease inhibitor cocktail (78415; Thermo Fisher Scientific). Protein quantification was performed using the Pierce™ BCA Protein Assay (23225 and 23227; Thermo Fisher Scientific). Mini-Protean^®^TGC™ wells were loaded with 5 μg of EV protein in Laemmli Sample Buffer (1610747; Bio Rad) or 10 μL of the Precision Plus Protein Kaleidoscope prestained standards (1610375; BioRad).

Gel electrophoresis was run, samples and standards were transferred to a nitrocellulose membrane, and the membrane was labeled for heat shock protein 70 (HSP70) or tumor susceptibility gene 101 (TSG101) (EXOAB-Hsp70A-1 and EXOAB-TSG101–1, System Biosciences). Appropriate secondary antibody was applied and stained using SuperSignal™ West Pico Plus Chemiluminescent Substrate (34580; Thermo Fisher Scientific). Images were taken with ChemiDoc™ XRS+ with Image Lab™ Software (BioRad).

### Isolation of peripheral blood-derived monocytes

Whole blood was mixed 1:1 with PBS and separated by density gradient centrifugation (Ficoll^®^ Paque Plus, 17-1440-02; Cytiva Life Sciences). The mononuclear cell layer was then collected, washed twice with PBS, and plated in a 96-well plate (167008; Fisher ThermoScientific) at 1 × 10^6^ cells/well in 200 μL RPMI-1640 for 2 h. Media containing nonadherent cells were removed to select adherent monocytes, and the remaining cells were gently washed with 100 μL PBS twice to remove additional nonadherent cells.

Adherent cells were cultured in RPMI-1640 with Abx and 10% fetal bovine serum (FBS; complete RPMI) with 10 ng/mL ovine macrophage colony-stimulating factor 1 (MCSF-1; RP1640G; KingFisher Biotech Inc.) for 7 days, with media refreshed on day 4. From day 7 onward, peripheral blood-derived monocytes were considered macrophages.^26,27^ Macrophage phenotype was confirmed by flow cytometry using established markers for CD205 (MCA2450PE), CD11b (MCA1425A647), CD45 (MCA2220B), or MHC class II (MCA2226F).^28^ All antibodies for flow cytometry were obtained from Bio-Rad. Conservative gating was performed for analysis using FlowJo software (Ashland, OR).

### Macrophage EV education

On day 8, macrophage culture media were replaced with 170 μL of complete RPMI and 30 μL of (1) PBS, (2) MSCdEVs, or (3) TdEVs with concentrations of 1 × 10^7^ particles/30 μL. After 24 h, the media were again replaced with the same treatments and serum-free RPMI. After an additional 24 h, the media were collected, and cell debris was removed by centrifugation. Supernatants were aliquoted and stored at −20°C. The cultured wells (*n* = 24) thus comprised three cell lines, each derived from a different animal, which were given one of three treatments in technical duplicate.

### Multiplex immunoassay of EV-educated macrophages

Educated macrophage-enriched media were analyzed using a commercially available enzyme-linked immunosorbent assay kit (MILLIPLEX MAP KIT, ovine cytokine/chemokine and growth factor magnetic bead panel; Catalog No. SCYT1–91K-PXBK14; EMD Millipore Corp, Billerica, MA). Frozen banked media were slowly thawed to room temperature and processed undiluted in technical duplicate. The bioassay measured concentrations of IL-1α, IL-1β, IL-4, IL-6, CXCL8, IL-10, IL-17A, IL-36RA, IP-10, MIP-1α/CCL3, MIP-1β/CCL4, TNF-α, IFN-γ, and VEGF-A. All experimental wells were run in technical duplicate. Mean fluorescent intensity of mean of technical duplicates and experimental technical duplicates was used for statistical analysis (two technical replicates, one from each experimental group, were trimmed due to low bead count per the manufacturer's recommendations).

### Tenocyte migration and bioactivation

Tenocytes from passage five were plated in a 96-well plate (ImageLock, 4379; Essen Bioscience) at a density of 20,000 cells/well and grown to confluence. The first 24 of 48 h of this were in CD, while the second 24 h had the addition of 30 μL of PBS (negative control), MSCdEVs, or TdEVs with concentrations of 1 × 10^7^ particles/30 μL. After confluence, a uniform scratch was made in each well (WoundMaker™, Essen BioScience, Ann Arbor, MI).

Each well was then washed twice with 150 μL PBS. Cells were then cultured in 200 μL of CD (positive control) or 170 μL DMEM:F12 with Abx and 30 μL of PBS (negative control), MSCdEVs, or TdEVs with concentrations of 1 × 10^7^ particles/30 μL. Serial temporal images were taken of each well using an automated plate scanner (IncuCyte™, Essen BioScience) at 0 and 2 h, and every 6 h after that for a total of 20 h. Image analysis was used to measure the percent change in area of the decellularized scratch.^29^

### EV fluorescent labeling and uptake imaging

Purified EVs were stained using Vybrant™ DiD (V22887; Thermo Fisher Scientific) lipophilic dye and washed twice in PBS using ultracentrifugation. PBS with 10% exosome-depleted FBS (A2720801; Thermo Fisher Scientific) was treated and “stained” in parallel as a negative control. Macrophages were differentiated and cultured on glass coverslips (C8–1.5H-N; Cellvis). Cells were dosed with 30 μL of “stained” controls or stained EVs (1 × 10^7^ particles). Macrophages were then washed twice with PBS, fixed in 4% paraformaldehyde for 10 min, and stained with ActinGreen™488 ReadyProbes™ reagent (R37110; Thermo Fisher Scientific), and then with DAPI (EN62248; Thermo Fisher Scientific) before preservation in antifade mounting media (P36961; Thermo Fisher Scientific). Wells were then imaged using a spinning disc confocal microscope (IX83 P2ZF using cellSens Dimension v4.1, Olympus).^30^

### Statistical analysis

Scratch-wound confluence data were evaluated using multiple Mann–Whitney tests with significance considered for treatment and time factors where *p* < 0.05 and *q* < 0.05. Multiplex immunoassay mean fluorescent intensity was compared across experimental and control groups. To compare the overall difference between control and experimental groups, a two-way analysis of variance (ANOVA) was used with significance considered when *p* < 0.05. To compare shifts in cytokine levels between experimental groups, a multiple paired *t*-test analysis was performed with data paired by the donor. Significant discoveries were considered for those with q and *p*-values <0.05.

## Results

### EV characterization

**FIG. 1.**
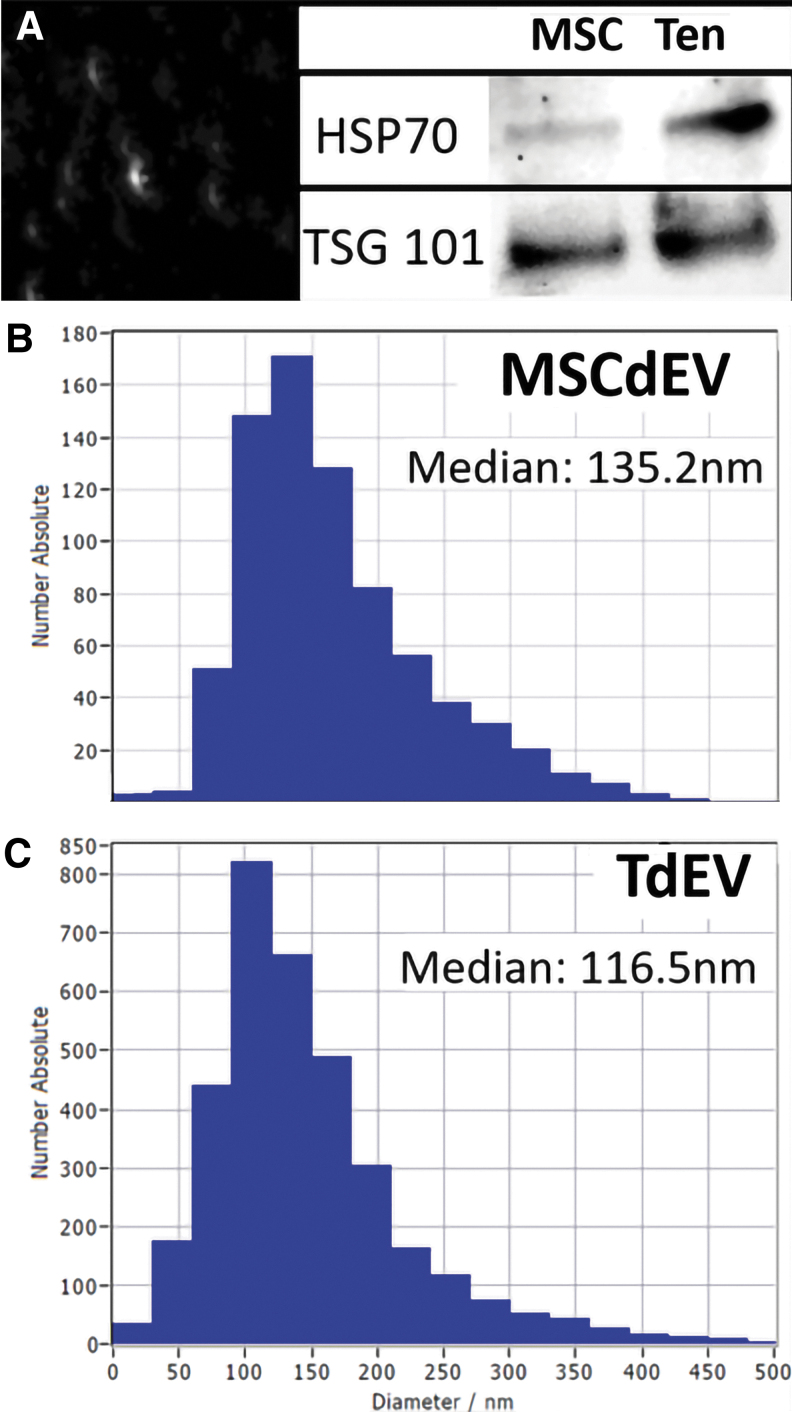
Differential protein expression in MSCdEVs compared to TdEVs of cell-enriched medium filtrates after size exclusion chromatography. **(A)** Representative photomicrograph of nanoparticles and Western blots demonstrating low, but present heat shock protein 70 (HSP70) content in EV lysates of MSCdEVs compared to greater expression in TdEVs with similar expression levels of TSG 101. **(B, C)** Histograms of the nanoparticle tracker analysis demonstrating the distribution of nanoparticles by size with median diameters reported.

Harvested EVs were assessed for purity and consistency. Brownian motion analysis demonstrated a median particle size of 116.5 nm (Standard deviation [StDev] 72.9 nm) for TdEVs and 135.2 nm (StDev 76.7 nm) for MSCdEVs (Fig. 1 A–C). These size ranges are consistent with exosomes being the predominant EV type present in these cell culture supernatants.^31^ Consistent with this observation, immunoblot analysis of purified TdEVs, and MSCdEVs, for the exosome-associated proteins HSP70 and TSG101 demonstrated positive immunoreactivity for a protein band at the expected molecular weights (70 and 44 kDa, respectively; Fig. 1A). HSP70 has been found to preferentially localize to the surface of exosomes,^32^ while TSG101 is considered an exosome marker due to its association with the endosomal sorting complex required for transport.^33^ These data confirm pure populations of EVs from each cell source.

### *In vitro* assessment of macrophage uptake of EVs

**FIG. 2.**
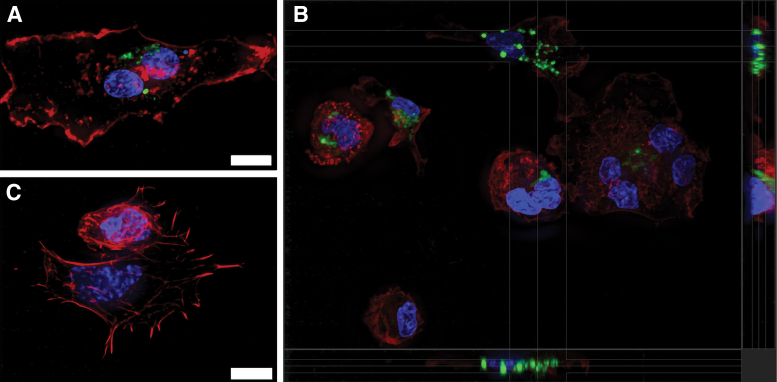
Spinning disc confocal photomicrographs of cultured macrophages stained with fluorescent markers for actin (*red*, phalloidin) and nucleic acids (*blue*, DAPI). **(A, B)** Macrophages cultured for 4 h with TdEVs that were lipid membrane labeled using DiD (*green*). **(A)** is a flattened z-stack view of a single, binucleate cell, while **(B)** is an expanded slice view of a field containing multiple macrophages with orthogonal views on the *bottom* and *right* sides of the image rendered from the z-stack and showing that the *green*, TdEVs, are within the cell body. **(C)** is a representative flattened z-stack view of control macrophages cultured for 4 h with 10% exosomes-depleted FBS in PBS that underwent the same staining process as the TdEVs used in **(A, B)**. All images are taken using a 60X oil immersion objective with synchronized exposures (*white* scale bars = 10 μm). FBS, fetal bovine serum; PBS, phosphate buffered saline.

Next, we sought to determine, *in vitro*, if macrophages represent a viable cellular target of endogenously released or exogenously administered EVs in the tendon microenvironment. Peripheral blood monocyte-derived macrophages were treated with fluorescently labeled EVs for 4 h; punctate fluorescent aggregates localized within the cytoplasm, consistent with EV uptake, based on counterstaining for actin and nucleic acid (Fig. 2A–C).

### Macrophage EV education

**FIG. 3.**
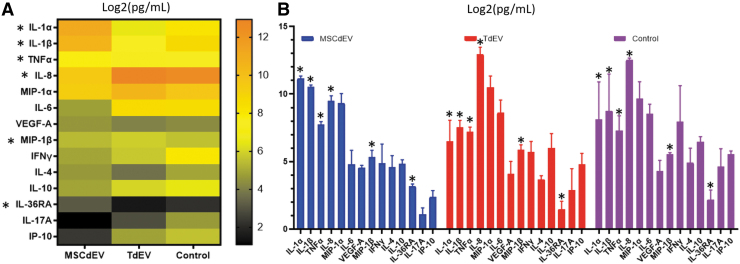
The mean accumulated cytokine levels expressed by macrophages over 24 h that were educated over 48 h by two doses of 1 × 10^7^ particles of either TdEVs or MSCdEVs in PBS or PBS without EVs, given 24 h apart. Cytokine expression was measured by multiplex bead-based immunoassay and reported as picogram per milliliter log2 to best visualize relative shifts in each analyte on a single scale. **(A)** A heat map highlighting the relative differences among treatment groups. **(B)** Bar graphs with standard deviations to highlight trends among treatment groups. The interaction of treatment with cytokine showed a significant difference between treatment groups (two-way ANOVA: *p* < 0.0001). *Asterisks* (*) mark the cytokines that demonstrate statistically significant differences between experimental groups; Multiple paired *t*-tests of the untransformed MFI, *p* < 0.05, *q* < 0.2. ANOVA, analysis of variance; MFI, mean fluorescent intensity.

**FIG. 4.**
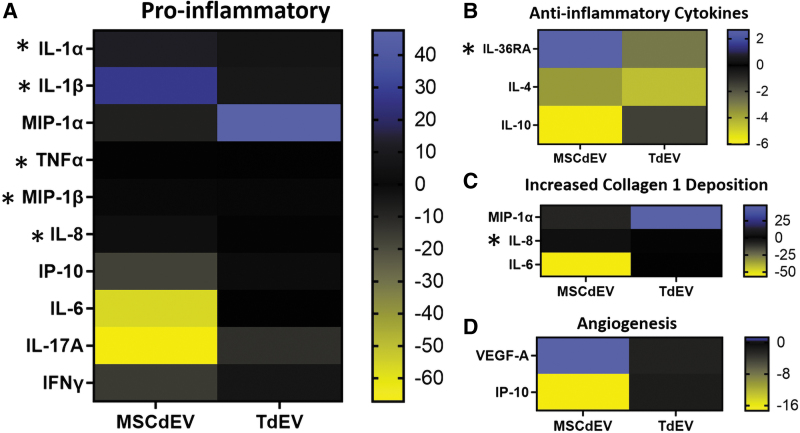
Heat map of cytokine expression as mean fluorescent intensity fold change of experimental groups relative to the control group separated by relevant functions. **(A)** highlights cytokines described as proinflammatory in the literature, **(B)** as anti-inflammatory, **(C)** as increasing collagen type 1 deposition, and **(D)** as driving angiogenesis. Statistical analysis was performed by multiple paired *t*-tests; cytokines marked by *asterisks* (*) have *p* and *q* values <0.05.

The impact of EV uptake by macrophage cytokine expression over 24 h was assessed. There was a significant difference in cytokine expression between both treatment groups and the negative control (*p* < 0.001; two-way ANOVA). Between the two treatment groups, there were several significant discoveries of a difference in cytokine expression (Table 1. multiple paired *t*-tests, *p* < 0.05, *q* < 0.05). Macrophages educated with MSCdEVs had higher expression of IL-1α, IL-1β, TNFα, CXCL8, and IL-36RA, whereas TdEV-educated macrophages had higher expression of MIP-1β/CCL4. These shifts in expression were relative to the negative controls and to each other, as demonstrated by mean picogram per milliliter (Fig. 3A, B) and when grouped by biological function and demonstrated by fold change in mean fluorescent intensity of experimental groups compared to the control groups (Fig. 4A–C).

**Table 1.** Statistical Analysis of the Cytokine Multiplex Immunoassay Performed Using Multiple Paired *t*-Tests. Note the Higher *p*-Values Among the Anti-Inflammatory Cytokines Compared to Many Proinflammatory and Collagen Deposition Driving Cytokines

Discovery	Analyte	*p*-	*q*
Yes	IL-1α	0.003	0.018
	IL-1β	0.006	0.018
	IL-36RA	0.007	0.018
	MIP-1β	0.007	0.018
	TNFα	0.012	0.023
	IL-8	0.017	0.027
No	IP-10	0.094	0.129
	IL-6	0.156	0.187
	IL-10	0.256	0.256
	IL-4	0.302	0.256
	IL-17A	0.315	0.256
	MIP-1α	0.321	0.256
	IFNγ	0.626	0.462
	VEGF-A	0.875	0.600

### Tenocyte migration

**FIG. 5.**
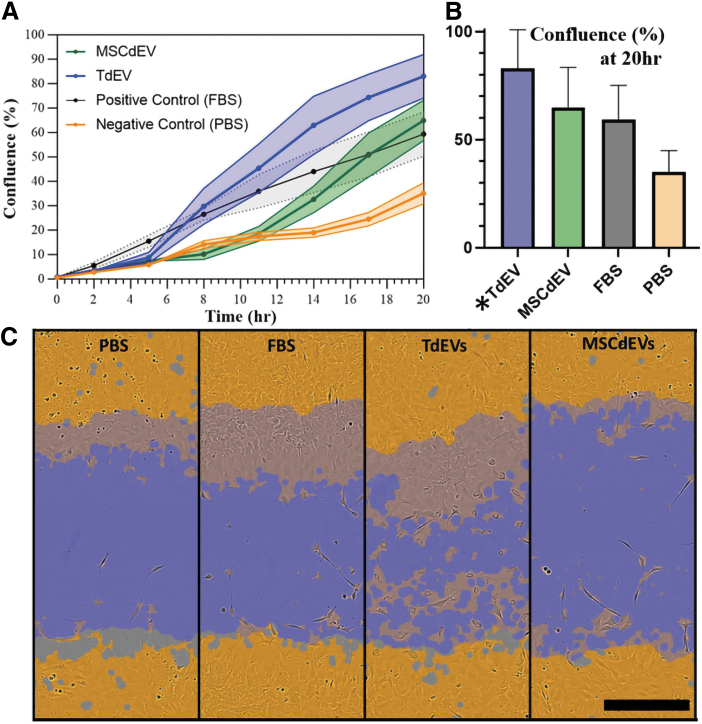
Tenocyte bioactivity measured by *in vitro* scratch-wound. Wounds were made in confluent cultures 24 h after experimental dosing with PBS, FBS, or 1 × 10^7^ particles of TdEV or MSCdEV in PBS, with a second dose administered after scratch-wound at time zero. **(A)** Percent confluence over time of each treatment group with shaded areas accounting for standard error. **(B)** relative percent confluence at 20 h with standard error. *Asterisks* (*) denote statistical significance compared to the negative control using multiple Mann–Whitney tests. **(C)** Representative wells of tenocyte cultures at time point zero, and the reflected same region at 11 h, highlighting the time point of most significant difference among groups. *Blue* highlights the initial scratch-wound, while *orange* highlights the cell profiles, and *gray* where cell profiles were absent outside the scratch-wound.

Finally, to assess whether tenocyte bioactivity was affected by treatment with TdEVs versus MSCdEVs, an *in vitro* scratch-wound analysis was performed to evaluate cell migration (Fig. 5 A–C). When treated with TdEVs, scratch-wound closure was, on average, 82.5% over 20 h. This closure rate and magnitude were significantly greater than for those tenocytes treated with MSCdEVs (64.9%), the positive, FBS-enriched controls (59.3%), or the negative, PBS controls (35.0%) (two-way ANOVA, treatment factor over 20 h: *p* = 0.0056; Fig. 5A, B).

## Discussion

As previous studies have demonstrated that paracrine signaling of MSCdEVs may be responsible for the positive therapeutic effects of AdMSCs on tendon healing,^12,20^ we sought to determine how TdEVs may differentially effect cellular responses associated with tendon healing using donor-derived tenocyte and macrophage *in vitro* cell culture models. We successfully *ex vivo* cultured and isolated EVs from donor ovine-derived tenocytes and AdMSCs. These EVs demonstrated similar size and appropriate protein expression and are bound and taken in by macrophages. Most notably, TdEVs increase tenocyte migration significantly more than MSCdEVs, and preferentially upregulate macrophage secretion of cytokines responsible for collagen type 1 deposition and crosslinking, favor a MIP1 and CXCL8 inflammatory profile, and decrease IL-1 secretion.

To evaluate the direct effect of MSCdEVs on tenocytes compared to TdEVs, we assessed cell migration using a scratch-wound assay. This simulates a tendon tear to resolve the cells' abilities to close this “wound.” We observed a significantly greater response of tenocytes to TdEVs compared to MSCdEVs, or the positive and negative controls (Fig. 5), suggesting greater upregulation of tenocyte bioactivity from exosomes that are autologous to their target cell. Notably, TdEVs more quickly began closing the “wound” and by 11 h had exceeded the positive control, which MSCdEVs did not match until the 17th h. There was a delay in the EV-treated groups' “wound” closure compared to the positive control of 5–8 h for TdEVs and 12–14 h for MSCdEVs.

This could be due to the protracted, but significant effect of gene regulatory signaling that vesicular cargo can play and the time for vesicular uptake and cargo integration compared to the more direct signaling of growth factors and other proteins present in FBS. It should be noted that no mitosis inhibitor, such as mitomycin C, was used, and thus, these data do not explicitly demonstrate the sole effect of EVs on migration. In addition, while these data are compelling, the suggested “autocrine” effect observed in this study does not fully recapitulate the complexity of paracrine signaling that is expected to take place *in vivo*.

As macrophages are one of the most abundant recruited cell types during early tendon injury and persist the longest in tendon repair, their phenotypic state is known to influence tendon healing. We sought to evaluate the effects of MSCdEVs compared to TdEVs on macrophage cytokine secretion. We observed differential secretion of multiple cytokines relevant to tendon healing when comparing macrophages educated with TdEVs compared to MSCdEVs. IFN-γ, IL-1β, and IL-1α drive M1 macrophage polarization,^34–37^ and IL-1β and TNFα have been reported to be increased in animal models of tendon injury, wherein they drive elevated matrix metalloproteinase (MMP) activity and progressive tendon degeneration.^34–36,38^ Our data showed 24-, 7.9-, and 1.46-fold increases in IL-1α, IL-1β, and TNF α, respectively, in macrophages educated with MSCdEV compared when educated using TdEVs (*p* = 0.006, 0.275, and 0.012, Figs. 3 and 4). IFN-γ showed a trend of strong downregulation of >7-fold by both EV treatments (*p* = 0.626).

In contrast, although not statistically significant, MIP-1α/CCL3 and IL-6 increased 2.3- and 14-fold, respectively, with TdEV treatment compared to MSCdEV (*p* = 0.32 and 0.156). MIP-1α/CCL3 not only drives fibroblast bioactivity but also is crucial for collagen type 1 production, especially in early tendon healing.^39,40^ Similarly, IL-6, while active in proinflammatory cascades in some tissues, plays multiple roles in tendon healing, including upregulation of IL-10 and suppression of TNF-β and IL-1β.^41^ It also plays a crucial role in tendon healing and integrity, possibly through its effects on fibroblast recruitment and collagen type 1 synthesis.^41–43^

IL-17A can further increase tenocyte expression of inflammatory cytokines, while increasing collagen type III expression and apoptotic factors.^44^ While not significantly different between the treatment groups, there is a greater than threefold reduction in expression in both treatment groups compared to the negative control, which may be favorable for tendon healing (Fig. 4).

Cytokines such as IP-10, MIP-1β/CCL4, and CXCL8 can act directly to recruit myeloid cells such as neutrophils and monocytes. Some of these similar molecules and others like VEGF-A are proangiogenic, increasing endothelial cell proliferation and neovascularization. These changes in the local cytokine milieu can result in significant changes within the cellular composition of the injured tendon microenvironment.^41,45–47^ Interestingly, we observed a trend in TdEV promoting IP-10 (5.2-fold; *p* = 0.094) expression, concurrent with suppressing VEGF-A (0.82-fold; *p* = 0.875) relative to MSCdEVs.

In addition, there was a significant increase in MIP-1β (1.4-fold) and CXCL8 (>10-fold) in macrophages treated with TdEVs compared to MSCdEV treatment (*p* = 0.007 and 0.017). Taken together, these data suggest a shift away from an angiogenic, proinflammatory, and collagenolytic phenotype in MSCdEV-educated macrophages toward a TdEV-driven phenotype that may be more conducive to early tendon repair in TdEV-educated macrophages, characterized by cytokine secretion that would further enhance monocyte and neutrophil chemotaxis, and promote collagen type 1 deposition and some angiogenesis.^41^

Anti-inflammatory cytokines IL-4 and IL-10 are both drivers of M2 polarization.^48–50^ IL-10 *in vivo* data show improved healing in the tendons of a mouse model with induced overexpression of IL-10. At the same time, increased concentrations of IL-4 correlate with increased collagen type 1 expression and cell proliferation in injured rotator cuff tendon.^46,48^ Interestingly, these data show trends in IL-10 expression increasing 2.2-fold with TdEV treatment over MSCdEVs (*p* = 0.256), while IL-4 expression is increased with MSCdEV treatment by 1.9-fold (*p* = 0.302). Another anti-inflammatory cytokine assessed, IL-36 receptor antagonist (IL-36RA), showed a 3.2-fold increase with MSCdEV treatment compared to TdEV (*p* = 0.007); however, the role of this cytokine in tendon homeostasis and healing is not understood yet.^51^

Some paracrine signaling may, in turn, influence exosome characteristics. IFN-γ was shown to prime mesenchymal stromal cells *in vitro* toward expression of exosomes that drive local anti-inflammatory effects in an *in vivo* mouse model.^37^ The effector molecules are not known and may, in-fact, be multi-model. Current research is beginning to elucidate some of these effector molecules, such as HSP70. This protein is preferentially expressed on exosomes where the concentration may vary. In an *in vivo* study, an increased HSP70 expression on exosomes was correlated with decreased cardiac fibrosis and *in vitro* decreased fibroblast bioactivity.^52^ This study was carried out using cardiac myofibroblasts and serum-derived exosomes.^52^ In our research, we see increased tenocyte migration as a metric of bioactivity in those cells treated with TdEVs, which demonstrated increased HSP70 expression (Fig. 1). This differential role of HSP70 warrants further investigation.

Ovine cell lines were used to demonstrate *in vitro* exosome function in a translational model of rotator cuff disease, that is, RCT.^53^ While the ovine model is excellent for translatable anatomy, mechanics, and surgical approach, antibody-based assays are limited in this species, especially toward exosome-specific markers.^53^ This limited the analyses that could be performed at the protein level and informed the choice of protein markers. In the macrophage education and scratch-wound experiments, one biological replicate of EV source was used due to the limited, biologically paired EV sample size. This similarly limited sample size. Future work will investigate the constituent variance of EV cell source and biological source and the direct effect of educated macrophages on tenocyte bioactivity. *In vivo* experimentation in this translatable model will be investigated as the therapeutic potential of TdEVs is established.

This insight into the influence of TdEVs on macrophage signaling is exciting. It demonstrates a shift in proinflammatory cytokines away from the IL-1 predominance seen in MSCdEV-educated macrophages and toward MIP-1 & CXCL8 predominance. Furthermore, there is an increased bias toward collagen synthesis and tenocyte bioactivity with decreased angiogenic signaling when TdEVs are used in place of MSCdEVs. These *in vitro* data demonstrate a compelling reason to further investigate the differential influence of exosomes by cell source on tendon healing and how control of exosome composition may lead to effective therapies for these difficult-to-treat tissues.

## Supplementary Material

Supplemental data

## Data Availability

Essential data to this article may be made available on reasonable request to the corresponding author.
