# Supplementary figures and images for: Exosome Cell Origin Affects In Vitro Markers of Tendon Repair in Ovine Macrophages and Tenocytes
Source: Tissue Eng Part A. 2023 May 10;29(9-10):282–91. doi: 10.1089/ten.tea.2022.0185 (PMC10178933; doi:10.1089/ten.tea.2022.0185)

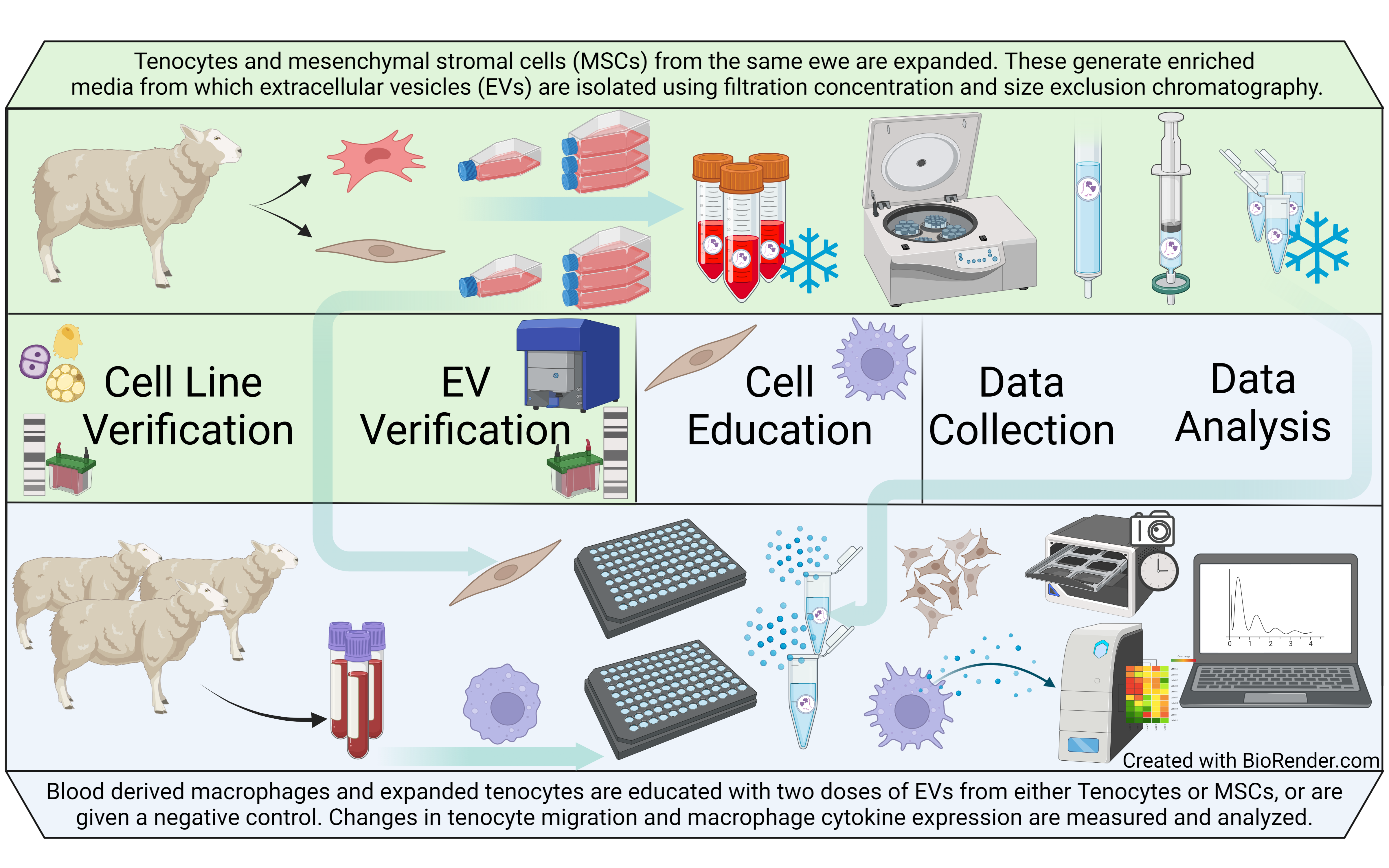

Supplement: Supplemental data [file Suppl_FigS1.jpg]
